# Machine Learning-Based Pipeline for High Accuracy Bioparticle Sizing

**DOI:** 10.3390/mi11121084

**Published:** 2020-12-07

**Authors:** Shaobo Luo, Yi Zhang, Kim Truc Nguyen, Shilun Feng, Yuzhi Shi, Yang Liu, Paul Hutchinson, Giovanni Chierchia, Hugues Talbot, Tarik Bourouina, Xudong Jiang, Ai Qun Liu

**Affiliations:** 1 ESIEE, Universite Paris-Est, CEDEX 93162 Noisy-le-Grand, France; shaobo.luo@univ-paris-est.fr (S.L.); giovanni.chierchia@esiee.fr (G.C.); 2 Nanyang Environment and Water Research Institute, Nanyang Technological University, Singapore 637141, Singapore; ktnguyen@ntu.edu.sg; 3 School of Mechanical & Aerospace Engineering, Nanyang Technological University, Singapore 639798, Singapore; 4 School of Electrical & Electronic Engineering, Nanyang Technological University, Singapore 639798, Singapore; shilun.feng@ntu.edu.sg (S.F.); YZShi@ntu.edu.sg (Y.S.); yang.liu@ntu.edu.sg (Y.L.); 5 Institute of Microsystem and Information Technology, Chinese Academy of Sciences, Beijing 200050, China; 6 Life Sciences Institute, National University of Singapore, Singapore 117456, Singapore; lsipeh@nus.edu.sg; 7 CentraleSupelec, Universite Paris-Saclay, 91190 Saint-Aubin, France; hugues.talbot@centralesupelec.fr

**Keywords:** machine learning, segmentation, CCD, CMOS, particle sizing

## Abstract

High accuracy measurement of size is essential in physical and biomedical sciences. Various sizing techniques have been widely used in sorting colloidal materials, analyzing bioparticles and monitoring the qualities of food and atmosphere. Most imaging-free methods such as light scattering measure the averaged size of particles and have difficulties in determining non-spherical particles. Imaging acquisition using camera is capable of observing individual nanoparticles in real time, but the accuracy is compromised by the image defocusing and instrumental calibration. In this work, a machine learning-based pipeline is developed to facilitate a high accuracy imaging-based particle sizing. The pipeline consists of an image segmentation module for cell identification and a machine learning model for accurate pixel-to-size conversion. The results manifest a significantly improved accuracy, showing great potential for a wide range of applications in environmental sensing, biomedical diagnostical, and material characterization.

## 1. Introduction

High accuracy size measurement is important for characterizing nanoscale and microscale particles. Various sizing measurement techniques are widely used to sort colloidal materials [1], analyze natural bioparticles such as pollen [2], characterize cells [3,4,5,6], examine soil particle [7], monitor food quality during harvest [8], and assess air quality [9]. For instance, a golden standard for monitoring parasites in drinking water systems identifies different types of bioparticles characterized by their sizes [5]. Commonly used particle sizing techniques include non-imaging-based sizing techniques such as sieve analysis [10], static laser light scattering [11], dynamic light scattering [12], nanoparticle tracking analysis [13], time-of-transition (TOT) principle [14], as well as imaging-based sizing techniques such as bright-field microscopy [15], fluorescent microscopy [16,17], and electron microscopy [18].

Sieve analysis [10,19] is a traditional method used to measure the particle size. It utilizes stacked sieves with increasing aperture sizes to clamp particles and generate a size distribution. Other non-imaging-based sizing techniques estimate the particle size indirectly. For instance, static laser scattering [11] measures the gyration size instead of the physical one based on the scattering pattern. Dynamic light scattering [12] retrieves the particle size based on the correlation function of scattered light signal, which essentially measures the diffusion coefficient of the particle. Nanoparticle tracking analysis [13] based on Brownian motion obtains the size from the diffusion coefficient of particle. The non-imaging-based sizing techniques mentioned above are unable to accurately determine the size of non-spherical particles due to the limit of applied models. Conventional flow cytometry [20] determines the size according to the scattering pattern or other optical signatures. Before that, the calibration using particles of known size is required. Unfortunately, the calibration is not generalizable because particles of the same size may have substantially different optical signatures due to the difference in materials, surface properties, internal structures, or fluorescent labels.

Imaging-based sizing techniques are able to provide a direct measurement of the physical size of particles based on image analysis. Image analysis is an automated method by using intelligent software to analyze results of a huge number of images. Images of microscale and nanoscale particles are usually acquired using imaging-based microscopy. Commonly used image sensors include single-point photodetectors such as photomultiplier tube (PMT) and avalanche photodiode (APD) [21] as well as two-dimensional (2D) photosensor arrays such as charge-coupled device (CCD) or complementary metal-oxide-semiconductor (CMOS) [22]. In a 2D sensing case, the size of individual particle is estimated by converting the pixel to size at a fixed conversion ratio which is determined theoretically according to the specifications of the optical components. For example, a single pixel in images taken corresponds to 0.33 μm with a 60× objective, and 0.5 μm with a 40× objective according to the product specifications. However, it is noticed that this fixed conversion ratio does not always give rise to an accurate particle sizing, probably arising from factors such as the objective error, imaging error, and segmentation error. Hence, the relationship between the pixel number and physical size is difficult to be modelled due to possible nonlinearity.

To address the issues mentioned above, a machine learning-based pipeline for imaging-based high accuracy particle sizing has been developed. The machine learning-based pipeline automatically segments micro particles from the images, estimates the pixel size of particles, and predicts the physical size from the pixel information using a machine learning model trained with labeled images of calibration spherical beads. Compared to conventional approaches, our intelligent pipeline offers a more accurate particle sizing by learning from the massive calibration data. This machine learning-enabled pipeline would greatly extend the applicability of imaging-based sizing in the field of environmental sensing, biomedical diagnostical, and material characterization.

## 2. Methods

**Figure 1:**
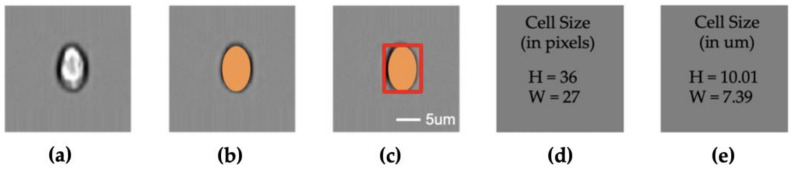
Size measurement pipeline; (**a**) cell image; (**b**) segmentation; (**c**) cell shape; (**d**) cell size in pixel numbers; (**e**) cell size in μm; (**a**–**c**) share the same scale bar.

The pipeline algorithm automatically analyzes the pixel information of the target particles and converts the pixel information into actual size based on a machine learning model (Figure 1a–e). First, it generates a contour of the particle using a segmentation algorithm. Then, the contour information is used to estimate the shape of the particle. Finally, the shape information is converted to physical length and width using the pixel-to-size module learnt by a quadratic machine learning model trained with least-squares regression [23] using the spherical beads of known sizes. All the aforementioned operations are integrated into an image processing pipeline to automatically predict the physical size of particles from images acquired using an imaging flow cytometry (Amnis^®^ ImageStream^®^X Mk II [24,25]).

### 2.1. Segmentation and Pixel Measurement Module

Deep learning has recently made impressive progress in imaging segmentation. For examples, U-Net [26], Deep Cell [27], Faster R-CNN [28], Mask R-CNN [29], and RetinaNet [30] have been demonstrated for instance segmentation in single cell analysis [31]. However, those deep learning models are computationally intensive and require heavy labelling from human. Imaging flow cytometry is capable of generating single cell image with a clear background. Hence it is well-suited for computer vision-based analysis.

**Figure 2:**
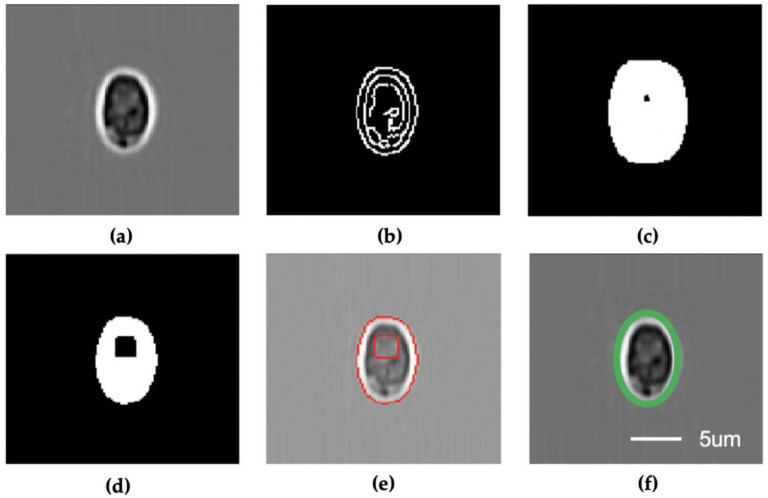
Segmentation and pixel measurement module. (**a**) Gaussian blur; (**b**) Canny detector; (**c**) Erode; (**d**) Dilate; (**e**) Find contours; (**f**) Estimate shape. All subfigures share the same scale bar.

The computer vision-based segmentation algorithm (Figure 2) first resizes the input single-particle images into 120 × 120 pixels and removes the noise using a Gaussian blurring module (Figure 2a). Then, a Canny detector is applied to the processed images to generate the edge images (Figure 2b) that are subsequently processed with erode (Figure 2c) and dilating (Figure 2d) algorithms to generate the output blob images. Next, the algorithm identifies the edge in the blob images and generates the contour information of the particle (Figure 2e). The height and width in terms of pixel numbers are estimated from particle contour (Figure 2f). In the case of spherical particles, the height and width have the same value. Finally, the physical size of the particles is determined based on the machine learning model.

Signal noise often degrades the image quality and introduces error to subsequent processing submodules in the pipeline. Gaussian blur [32] is a popular algorithm to reduce the noise and enhance the image quality. The formula of the Gaussian blur is expressed as

(1) $$g\left(x,y\right)=\underset{x,y}{\sum }f\left(x-i,y-j\right)h\left(i,j\right)$$

where $g\left(x,y\right)$ is the output pixel value, $f\left(x,y\right)$ is the input image pixel, and $h\left(i,j\right)$ is a Gaussian kernel given by

(2) $$h\left(i,j\right)=Aexp(-(\frac{{\left(i-i_{0}\right)}^{2}}{2\sigma_{i}^{2}}+\;\frac{{\left(j-j_{0}\right)}^{2}}{2\sigma_{j}^{2}}))$$

where A is the amplitude of the Gaussian kernel, $i_{0}$ and $j_{0}$ mark the center position of the kernel, and σ represent the standard deviation (SD) with respect to variables i and j.

Canny detector [33] is a popular technique in edge detection given its advantages in low error rate, high localizability, and minimized response. The Canny edge detection algorithm can be implemented by following steps:

Firstly, the gradient strength G and direction θ are calculated as

(3) $$G=\sqrt{G_{x}^{2}+G_{y}^{2}}\;\mathrm{and}\;\theta =\arctan\left(\frac{G_{y}}{G_{x}}\right)$$

where the $G_{y}$ and $G_{x}$ are the first derivatives of vertical direction ($G_{y}$) and horizontal direction ($G_{x}$), respectively. The θ is rounded to 0, 45, 90, or 135 degrees. For example, the θ in between 22.5 degree to 67.5 degree maps to 45 degree. Next, a non-maximum suppression algorithm is applied to remove non-considered pixel so that only the thin lines remain. Finally, a hysteresis stage with high and low threshold is applied on the lines to further improve the results.

Dilate and Erode [34] are two basic morphological operations for removing noise, isolating or jointing the individual components, and finding the intensity bumps or holes in an image. The dilate operation uses a kernel, such as 3 × 3 pixels, with an anchor point at the center of the kernel to scan over the image and calculate the maximum pixel value. That maximum value replaces the value in the anchor point. As a result, the bright regions are expanded, and the individual components with small gaps in between are connected. In contrast, the erode operation uses the minimal value to replace the value in the anchor point to render a thinner bright area.

The find contours operation [35] obtains the contour information. A contour is a closed curve where all its points are on the boundary and have the same value. In our algorithm, ellipse is used to approximate the outline of the cells. In the last stage of the imaging processing, the contour information of the cells is passed into an estimator function to obtain the inscribed rotated rectangle of the ellipse.

### 2.2. Size Converter Module

The size converter module converts the pixel to size in micrometers with the machine learning model. The calibration process started with the collecting images of microplastic beads with diameters in 3 μm, 4 μm, 4.6 μm, 5 μm, 5.64 μm, 7.32 μm, 8 μm, 10 μm, 12 μm, and 15 μm (from Thermo Fisher Scientific, Duke Scientific and Polysciences Inc., Warrington, FL, USA ). Then, they were processed with the segmentation algorithm to generate the beads diameters in pixels. Finally, a quadratic curve model was learned. The matrix equation regarding the least-square regression [36] parameters of *a*, *b*, and *c* in quadratic curve $y=ax^{2}+bx+c$ can be calculated by

(4) $$\left[\begin{array}{ccc} n & s_{1} & s_{2}\\ s_{1} & s_{2} & s_{3}\\ s_{2} & s_{3} & s_{4} \end{array}\right]\left[\begin{array}{c} c\\ b\\ a \end{array}\right]=\left[\begin{array}{c} z\\ \gamma_{yx}\\ \gamma_{yx^{2}} \end{array}\right]$$

where $s_{k}=\;\overset{n}{\underset{i=1}{\sum }}x_{i}^{k}$, $z=\;\overset{n}{\underset{i=1}{\sum }}y_{i}$, $\gamma_{yx^{j}}=\overset{n}{\underset{i=1}{\sum }}y_{i}x_{i}^{j}$. Inside the equation, $x_{i}$ is the pixels size of the individual bead and $y_{i}$ is the corresponding physical size of the bead, and n is the total number of beads.

When the linear models are learned, we obtained the parameters *m* = 0.2905 and *b* = 0.4785 for the linear model and *a* = −0.000163, *b* = 0.301, and *c* = −0.618 for the quadratic curve model. As the Root Mean Square Error (RMSE) of the quadratic model is smaller than the linear model (0.2657 vs 0.2668), the quadratic curve model was adopted to implement the size converter module.

### 2.3. Performance Evaluation

To evaluate the performance of the image processing pipeline, the image database of microplastic beads of known sizes and biological cells have been built. First, the image segmentation algorithm was evaluated with the Intersection over Union (IoU) metric [37]. Then, the performance of the machine learning model was evaluated with Root Mean Square Error (RMSE). Finally, the measurement on a realistic cell dataset was performed. The mathematical expressions of IoU and RMSE metrics are expressed as

(5) $$IoU=\frac{target\cap prediction}{target\cup prediction}$$

and

(6) $$RMSE=\sqrt{\frac{1}{n}\overset{n}{\underset{j=1}{\sum }}{\left(y_{j}\;-\;{\hat{y}}_{j}\right)}^{2}}$$

where target is the area in ground truth and prediction is the target segmented area; $y_{j}$ is the physical size, ${\hat{y}}_{j}$ is the predicted size and n is the total number of particles. Furthermore, the height and width distributions of particles such as beads, *Cryptosporidium* and *Giardia* oocytes are determined using bright-field imaging flow cytometry.

## 3. Results and Discussions

### 3.1. Segmentation and Pixel Measurement

**Figure 3:**
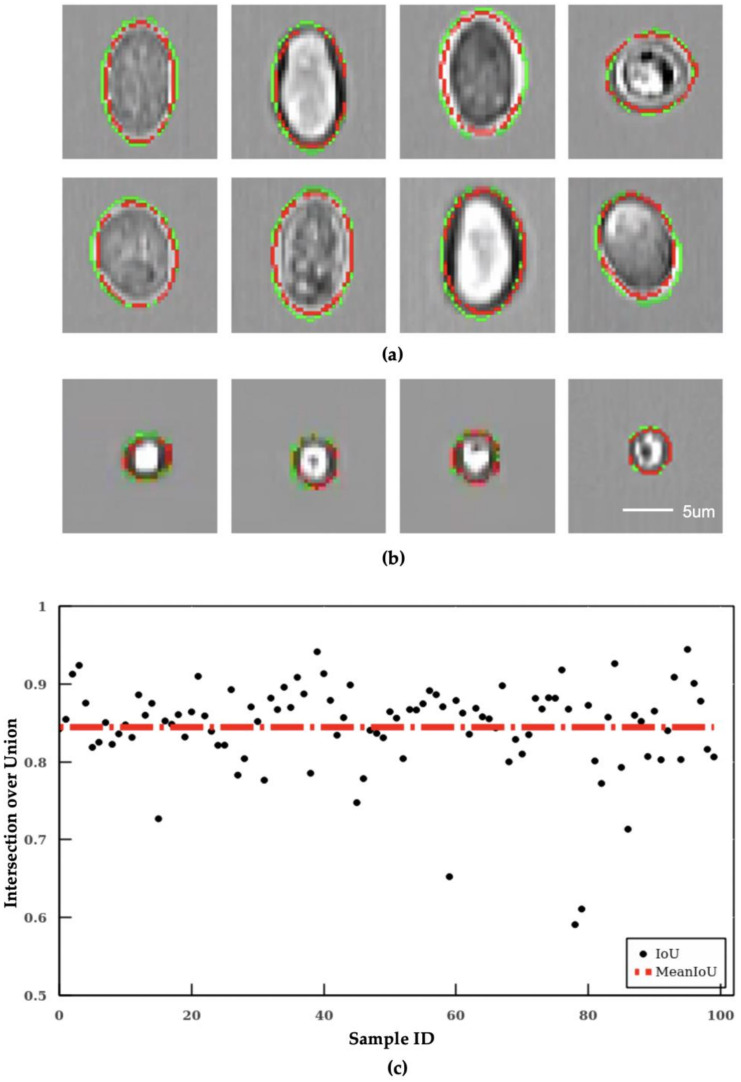
Error analysis on the segmentation algorithm and quadratic curve-based calibration. The image output of the segmentation algorithm of (**a**) segmentation results of *Giardia,* and (**b**) segmentation results of *Cryptosporidium*. All subfigures share the same scale bar. (**c**) intersection over Union results of the segmentation algorithm.

The segmentation results are evaluated with the IoU score between the contour labelled by human operators and the contour predicted by the algorithm. The output of the segmentation algorithm IS depicted in Figure 3a. The top two rows are the results of segmented *Giardia oocyte* images, and the lowest row is the results of segmented *Cryptosporidium oocyte* images. In these images, the green line is the ground truth (human labeled), and the red line is the output of the segmentation algorithm. As shown in Figure 3a, the image outputs of the segmentation algorithm are close to the ground truth. Overall, the segmentation algorithm achieved 84.4% in mean IoU (red dotted line) as shown in Figure 3b in which each blue dot represents the IoU of an individual image output of the testing dataset.

### 3.2. Physical Size Measurement

**Figure 4:**
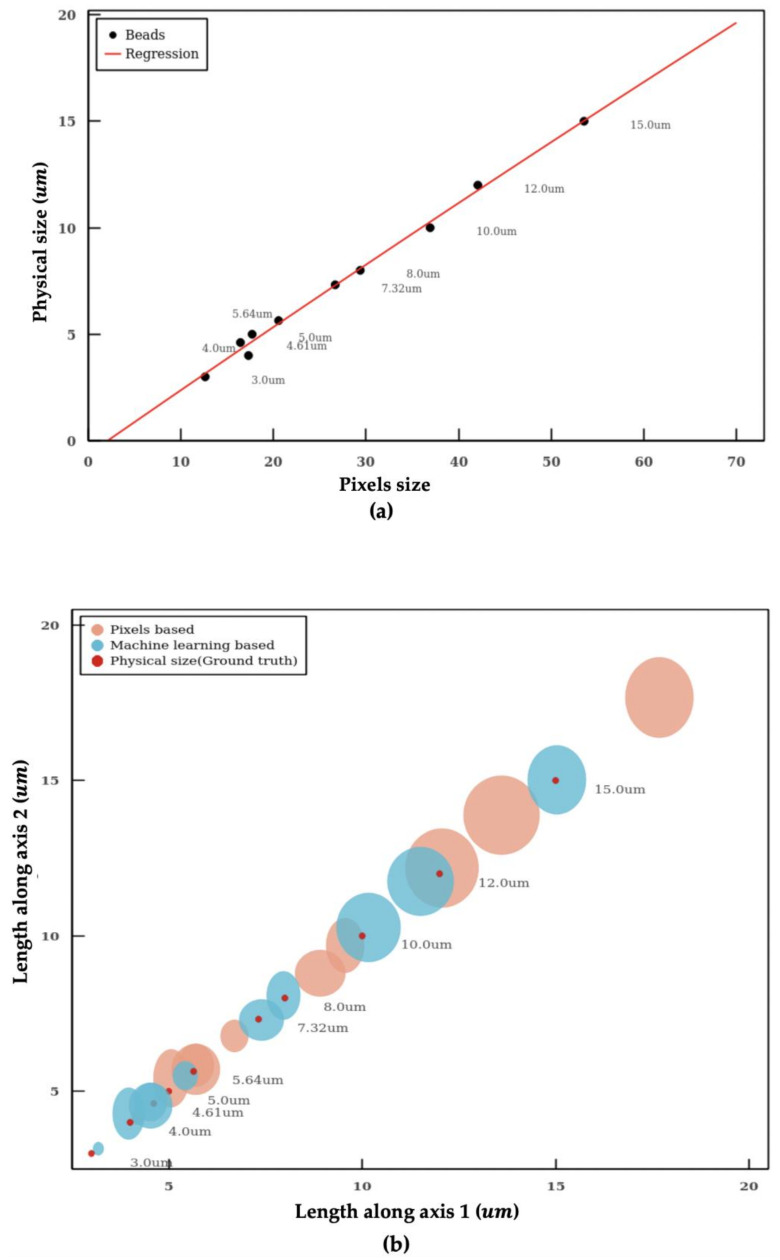
Calibration of size measurement. (**a**) quadratic curve-based calibration. (**b**) the length distribution in both axes of microplastic particles within a distribution range. The circle represents the population distribution with the error in 2σ range. Red colored ones are the physical diameters of beads, pink color ones are the beads sizes based on assumption, and cyan colored ones are the beads sizes based on machine learning calibration.

The imaging flow cytometer uses a fixed pixel-to-size ratio based on the specifications of the optics for particle sizing. However, this approach often leads to large errors in particle size (Table 1). Therefore, a machine learning model is established to determine the pixel-to-size ratio for accurate sizing. Both linear and quadratic regression models are adopted to learn the relationship between the pixel (pixels) and length (μm) of microplastic beads of known sizes. As the Root Mean Square Error (RMSE) of the quadratic model is smaller than the linear model (0.2657 vs 0.2668), the quadratic curve model was employed. Figure 4a shows the diameter versus the pixel size of the microplastic beads. The quadratic machine learning regression model is shown as the blue curve.

The sizes of the microplastic beads measured using our algorithm and using the fixed pixel-to-size ratio (0.33 μm/pixel with 60× objective on Amnis Imagestream MKII) are summarized in Table 1 and Figure 4b. The fixed pixel-to-size conversion ratio is the mainstream approach used by imaging flow cytometry. Our algorithm shows significantly more accurate sizing in comparison. Figure 4b shows the length distribution in both axes of microplastic particles within a distribution range. The red dots represent the actual sizes of the beads (ground truth based on manufacturer’s specifications), the brown dots present the size measured using the fixed pixel-to-size ratio, and the dark green dots represent the size measured using the machine learning model. The sizes of the dots represent the SD of the measurement. The microplastic particles have a narrow distribution with a CV < 2% according to product specifications.

The machine learning model gives rise to significantly more accurate size measurement compared to the approach using a fixed pixel-to-size ratio. The sizes of microplastic beads measured using the machine learning model deviate only slightly from the ground truth with a mean percentage error of 4.2% (Table 1). In contrast, the mean percentage error using the fixed conversion ratio is 23.3% which is five times larger than the machine learning model.

**Figure 5:**
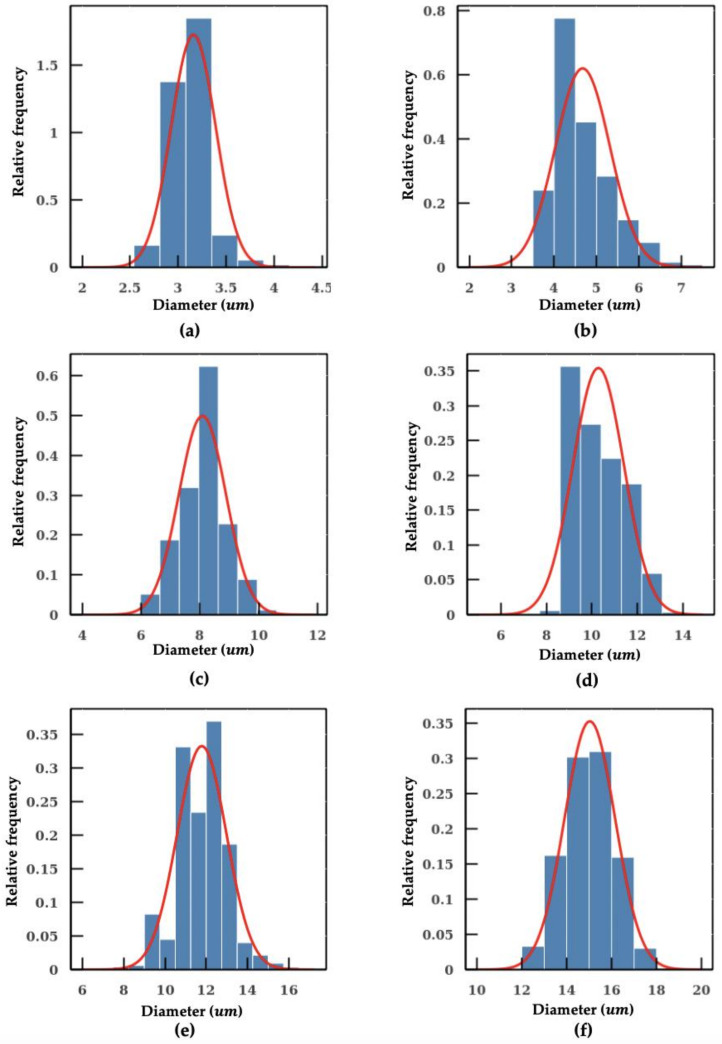
Measurement of individual microplastic particles sizes distribution: (**a**) 3 μm; (**b**) 5 μm (**c**) 8 μm; (**d**) 10 μm; (**e**) 12 μm; and (**f**) 15 μm.

**Figure 6:**
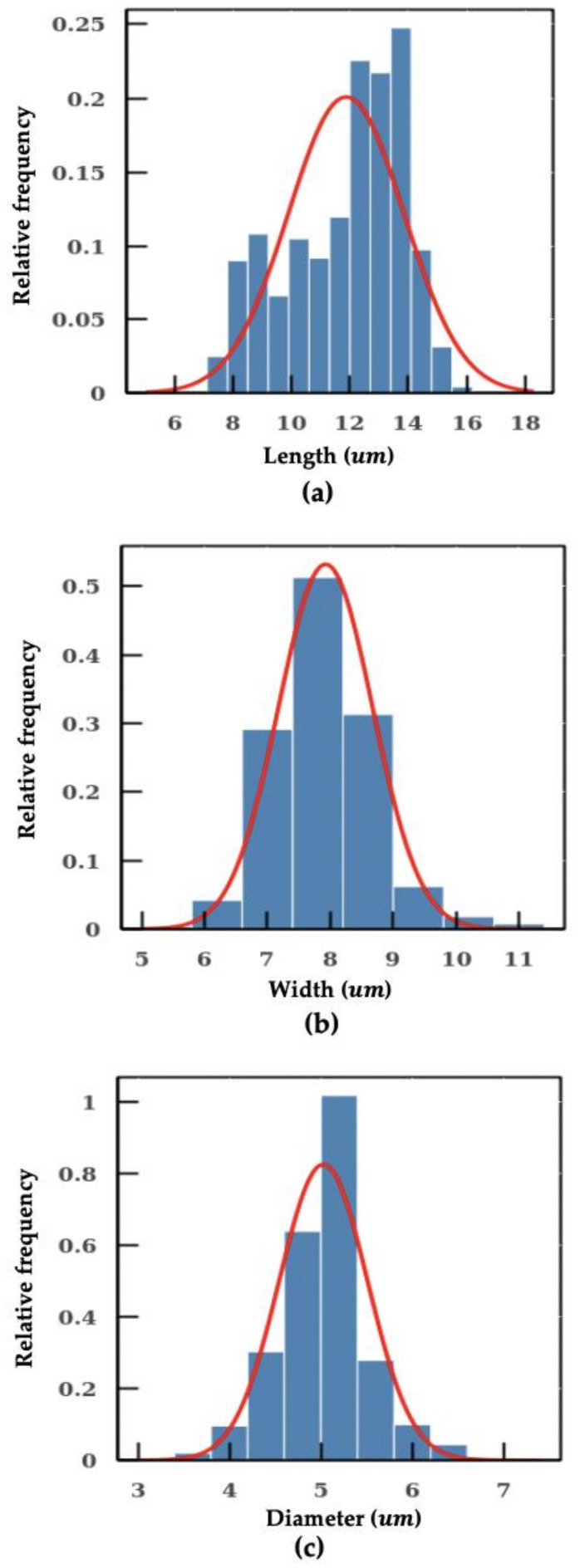
Measurement on *Cryptosporidium* and *Giardia*. (**a**) length of the *Giardia*. (**b**) width of the *Giardia*. (**c**) diameter of the *Cryptosporidium*.

As shown in Figure 4b, methods using fixed conversion ratio tends to overestimate the size of the particle. In the worst scenario, the percentage error even reaches a value close to 40%. In addition, the SD measured with the machine learning model is also smaller in comparison, which indicates a better precision in particle sizing. The individual measurements of microplastic beads of 3 μm, 5 μm, 12 μm and 15 μm using the machine learning model are shown in Figure 5. The aforementioned algorithms were integrated into a pipeline. With this intelligent pipeline, the height and width distributions of *Cryptosporidium* and *Giardia oocytes* are determined using bright-field images from imaging flow cytometry. The results are presented in Figure 6 and Table 2. Our intelligent pipeline determines that the mean height of *Giardia oocytes* is 11.87 μm with an SD of 1.9 μm. The mean width of the *Giardia oocytes* is 7.92 μm with an SD of 0.75 μm. The *Cryptosporidium oocytes* are approximately spherical, and the mean diameter *Cryptosporidium oocytes* measured using our algorithm is 5.03 μ with an SD of 0.48 μm. In contrast, the mean height and width of *Giardia oocytes* are 12.94 μm and 8.45 μm, and the mean diameter *Cryptosporidium oocytes* is 5.17 μm when calculated using the fixed conversion ratio.

## 4. Conclusions

In this paper, a machine learning-based pipeline for imaging-based high accuracy bioparticle sizing is demonstrated. It consists of an image segmentation module for extracting contours and estimating the pixel size of the bioparticle as well as a machine learning model for accurate pixel-to-size conversion. The image segmentation algorithm achieves 84.4% in the mean IoU, and the particle size determined by the machine learning model only has a mean percentage error of 4.2% which is five times better than the methods using a fixed pixel-to-size conversion ratio (23.3%). Our method empowers different intelligent imaging systems such as imaging flow cytometry for high accurate particle sizing and promises great potential for a wide range of applications in the field of environmental sensing, biomedical diagnostics, and material characterization.

## Figures and Tables

**Table 1:** Measurement error analysis.

Beads	Learnt Model			Fixed Ratio (0.33 μm/Pixel)		
	μ	σ	Error (%)	μ	σ	Error (%)
3.0 μm	3.15	0.21	5.1	4.16	0.24	38.8
4.0 μm	4.54	0.73	13.5	5.71	0.82	42.7
4.6 μm	4.28	0.83	7.1	5.42	0.93	17.6
5.0 μm	4.66	0.61	6.9	5.84	0.69	16.8
5.64 μm	5.50	0.46	2.5	6.78	0.51	20.2
7.32 μm	7.29	0.66	0.4	8.80	0.74	20.2
8.0 μm	8.08	0.77	1.0	9.69	0.88	21.2
10.0 μm	10.27	1.11	2.7	12.18	1.27	21.8
12.0 μm	11.75	1.10	2.1	13.89	1.27	15.7
15.0 μm	15.02	1.10	0.1	17.67	1.29	17.8
Avg.		0.76	4.2		0.86	23.3

**Table 2:** Measurement results on bioparticles.

	Feature	Mean (μm)	SD (μm)
*Giardia*	Height	11.87	1.99
	Width	7.92	0.75
*Cryptosporidium*	Diameter	5.03	0.48
